# High-fat diet-induced dyslipidemia drives retinal ECE-1 and ET-1 upregulation

**DOI:** 10.3389/fendo.2025.1640890

**Published:** 2025-08-29

**Authors:** Shuo Sun, Huilan Zhang, Jing Chen, Kaiwen Hei, Yi Hou, Yanhui Yang, Chunyan Shan, Longli Zhang

**Affiliations:** ^1^ Tianjin Key Laboratory of Retinal Functions and Diseases, Tianjin Branch of National Clinical Research Center for Ocular Disease, Eye Institute and School of Optometry, Tianjin Medical University Eye Hospital, Tianjin, China; ^2^ NHC Key Laboratory of Hormones and Development, Chu Hsien-I Memorial Hospital and Tianjin Institute of Endocrinology, Tianjin Medical University, Tianjin, China; ^3^ Tianjin Key Laboratory of Metabolic Diseases, Tianjin Medical University, Tianjin, China

**Keywords:** high-fat diet, endothelin-1, endothelin-converting enzyme-1, retina, dabetic retinopathy

## Abstract

**Background:**

High-fat diet (HFD) consumption is a major contributor to metabolic disorders, including obesity, dyslipidemia, and endothelial dysfunction, which have systemic and ocular consequences. Emerging evidence suggests that metabolic disturbances can lead to retinal pathology, but the underlying mechanisms remain unclear. Endothelin-1 (ET-1) and its regulatory enzyme, endothelin-converting enzyme-1 (ECE-1), play critical roles in vascular dysfunction. However, their involvement in HFD-induced retinal changes has not been fully elucidated.

**Methods:**

We used a mouse model of HFD-induced metabolic dysfunction and assessed systemic metabolic parameters, including lipid profiles, liver function markers, and inflammatory cytokines. Retinal gene expression of inflammatory and vascular factors, including ET-1 and ECE-1, was quantified using qPCR. Correlation analyses were performed to evaluate the relationship between systemic metabolic alterations and retinal molecular changes.

**Results:**

HFD feeding led to significant metabolic disturbances, including increased body weight, elevated total cholesterol (TC) levels, and hepatic stress. Retinal analysis revealed a significant upregulation of pro-inflammatory cytokines (IL-1β, IL-6, TNFβ1, and TNFSF15), as well as increased expression of ECE-1 and ET-1. Notably, correlation analysis demonstrated a strong positive association between TC levels and retinal ECE-1 (Pearson’s r = 0.888, p = 0.018*) and ET-1 (Pearson’s r = 0.815, p = 0.048*), suggesting a mechanistic link between systemic dyslipidemia and retinal vascular dysfunction.

**Conclusion:**

Our findings provide compelling evidence that HFD-induced dyslipidemia is associated with retinal inflammation and endothelial dysfunction, with ECE-1 and ET-1 serving as key mediators. These results highlight a potential therapeutic target for preventing retinal complications associated with metabolic disorders.

## Introduction

High fat diet (HFD) consumption has become a pervasive dietary pattern worldwide, contributing significantly to the rising incidence of metabolic disorders, including obesity, insulin resistance, and type 2 diabetes mellitus (T2DM) (1). These metabolic perturbations are intricately linked to systemic inflammation, dyslipidemia, and endothelial dysfunction, which not only affect major organs like the liver and cardiovascular system but also impact ocular tissues. In recent years, emerging evidence has underscored the impact of metabolic stress on the retina, leading to a spectrum of pathological changes that culminate in conditions such as diabetic retinopathy (DR) (2). DR is a leading cause of vision loss and is characterized by microvascular alterations, increased vascular permeability, and in advanced stages, neovascularization (3).

HFD consumption exerts profound effects on systemic lipid metabolism. Dyslipidemia, one of the hallmarks of metabolic syndrome, is characterized by altered levels of triglycerides (TG), total cholesterol (TC), high-density lipoprotein cholesterol (HDL-C), low-density lipoprotein cholesterol (LDL-C), as well as other markers such as aspartate aminotransferase (AST), alanine aminotransferase (ALT). These biomarkers provide valuable insights into the systemic metabolic status of an organism and can serve as surrogates for the severity of metabolic dysregulation (4).

Recent studies have suggested that metabolic dysregulation in HFD models can lead to significant alterations in retinal structure and function (5, 6). High fat feeding not only induces systemic metabolic abnormalities but also precipitates local retinal changes characterized by oxidative stress, inflammation, and endothelial dysfunction. A key molecular mediator in vascular pathology is endothelin-1 (ET-1), a potent vasoconstrictor peptide known for its pro-inflammatory and fibrogenic activity. ET-1 is synthesized from its inactive precursor by endothelin-converting enzyme-1 (ECE-1), making the ECE-1/ET-1 axis a central regulator of vascular tone, endothelial homeostasis, and inflammation (7, 8).Increasing evidence has implicated ET-1 in microvascular damage associated with diabetes, hypertension, and atherosclerosis. In the retina, ET-1 expression is elevated in diabetic retinopathy and contributes to endothelial dysfunction, capillary occlusion, and inflammatory signaling (9, 10). Furthermore, dyslipidemia—particularly elevated total cholesterol (TC)—has been shown to upregulate ET-1 in systemic vasculature (11), although its impact on the retinal endothelin pathway remains largely unexplored. Given these mechanistic links and the known role of ET-1 in other vasculopathic models, we hypothesized that HFD-induced dyslipidemia may activate the ECE-1/ET-1 axis in the retina, contributing to early microvascular dysfunction even in the absence of overt diabetes. To investigate this hypothesis, we used a HFD mouse model to systematically measure multiple blood parameters, while simultaneously quantifying the expression of ECE-1 and ET-1 in the retinas of the mice. By comparing the differences between the HFD and control groups and conducting correlation analyses, we further explored the relationship between abnormal blood parameters and retinal ECE-1 and ET-1 expression. This approach aims to uncover the potential association between systemic metabolic abnormalities and local retinal pathology.

## Results

### HFD induces obesity, dyslipidemia, and hepatic stress in mice

**Figure 1:**
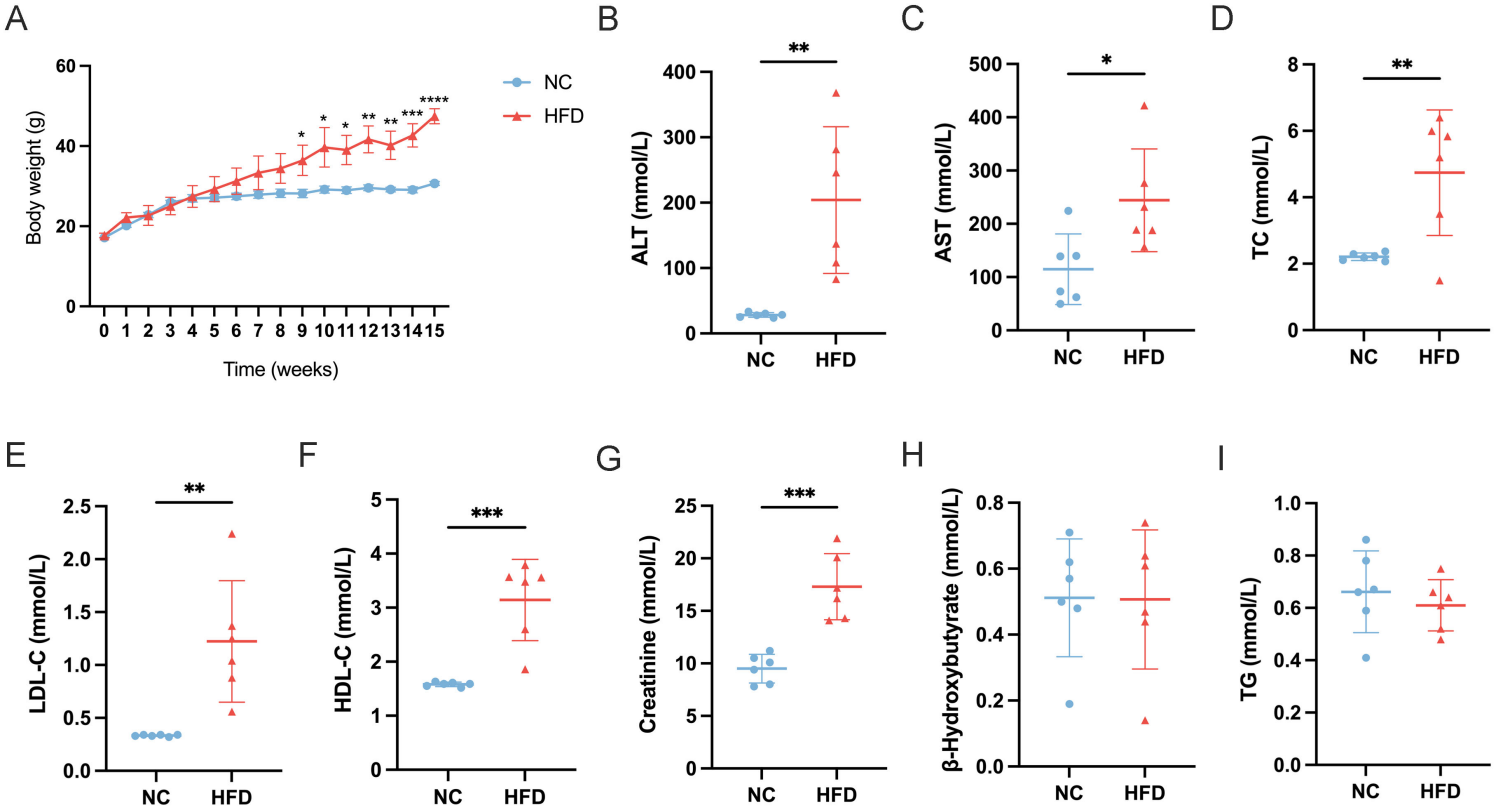
Effects of a high-fat diet **(HFD)** on body weight and serum biochemical parameters in mice. **(A)** Body weight progression over 15 weeks in normal chow (NC) and HFD-fed mice (n=6). **(B-I)** Serum levels of **(B)** alanine aminotransferase (ALT), **(C)** aspartate aminotransferase (AST), **(D)** total cholesterol (TC), **(E)** low-density lipoprotein cholesterol (LDL-C), **(F)** high-density lipoprotein cholesterol (HDL-C), **(G)** creatinine, **(H)** β-hydroxybutyrate, and **(I)** triglycerides (TG) in NC and HFD-fed mice. Data are presented as mean ± standard deviation (SD). Statistical significance was determined using an unpaired t-test. *p < 0.05, **p < 0.01, ***p < 0.001, ****p < 0.0001.

To establish a HFD‐induced metabolic disturbance model, we fed mice with either a normal chow (NC) diet or an HFD for 15 weeks and monitored body weight and multiple blood parameters. As shown in **Figure 1A**, HFD‐fed mice exhibited a progressive increase in body weight compared to NC‐fed controls, becoming statistically significant from approximately week 5 onward (p < 0.05). By the end of the experimental period, the average body weight in the HFD group was markedly higher than in the NC group, indicating successful induction of obesity.

In line with the increased body weight, liver function enzymes were significantly altered in HFD‐fed mice. Serum ALT levels (**Figure 1B**) were markedly elevated (p < 0.01) in HFD‐fed mice relative to controls, and AST levels (**Figure 1C**) also showed a significant rise (p < 0.05). These findings suggest hepatic stress or damage associated with excessive fat intake. Dyslipidemia was evident in the HFD group. TC levels (**Figure 1D**) were significantly higher in HFD‐fed mice compared to NC mice (p < 0.01), and LDL‐C concentrations (**Figure 1E**) similarly increased (p < 0.01). Interestingly, HDL‐C levels (**Figure 1F**) also rose (p < 0.001) in the HFD group, suggesting an overall disruption of normal cholesterol homeostasis rather than a simple shift in LDL‐C alone. Renal function and ketone metabolism indicators were also measured. Creatinine levels (**Figure 1G**) were significantly elevated in HFD‐fed mice (p < 0.001), indicating potential alterations in renal filtration or muscle metabolism under long‐term HFD exposure. In contrast, β‐hydroxybutyrate (B‐HB) levels (**Figure 1H**) did not differ significantly between the two groups, suggesting that these mice had not entered a pronounced ketotic state. Lastly, TG (**Figure 1I**) showed no significant difference between HFD‐ and NC‐fed mice, which may reflect a complex interplay between hepatic lipid production, peripheral uptake, and storage in adipose tissue.

Taken together, these results confirm the successful establishment of an HFD‐induced obesity model and demonstrate significant metabolic and hepatic perturbations, characterized by increased body weight, elevated liver enzymes, and dysregulated cholesterol profiles.

### HFD elevates retinal inflammation and vascular dysregulation in mice

**Figure 2:**
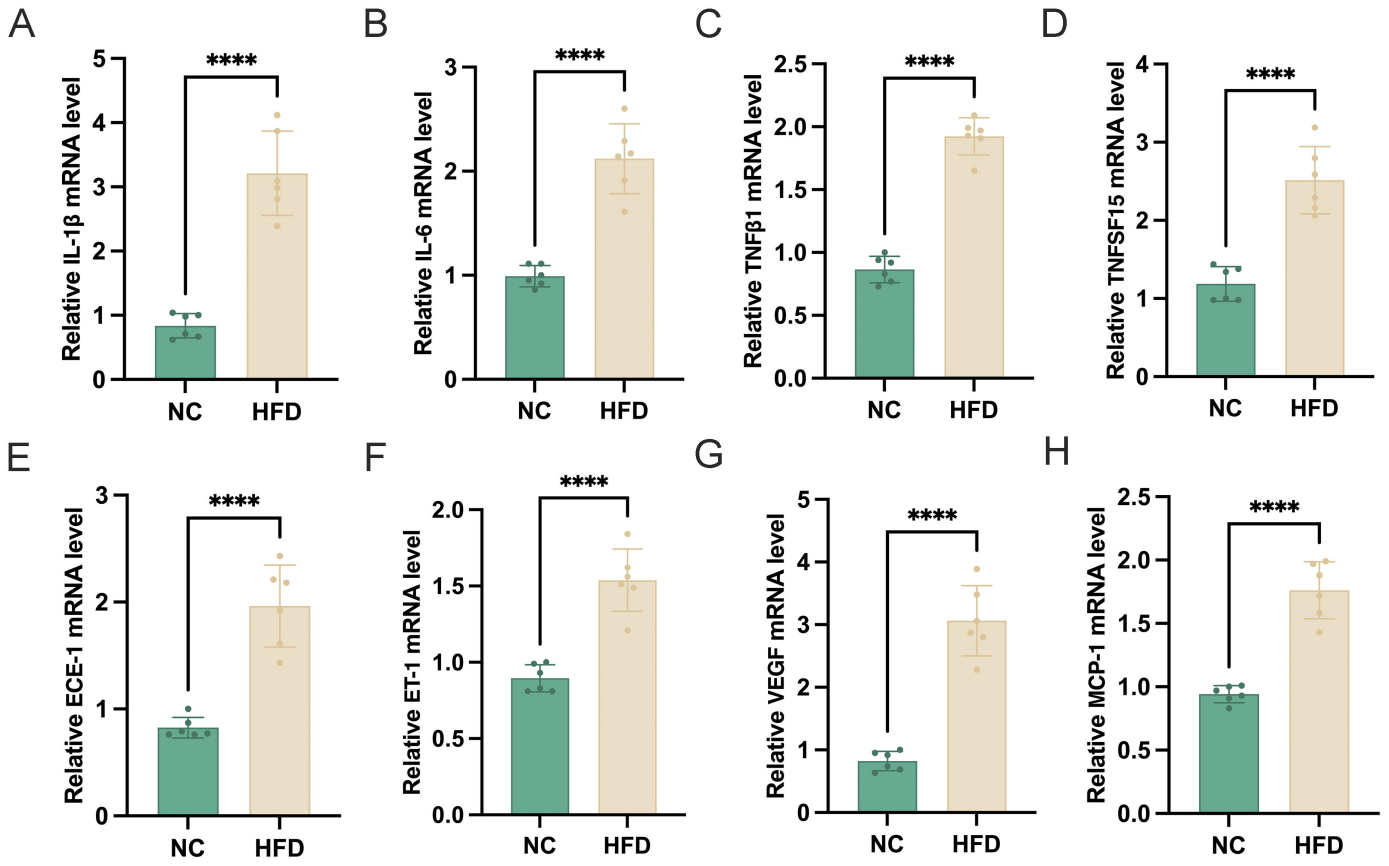
Effects of a high-fat diet **(HFD)** on retinal mRNA expression of inflammatory and vascular-related genes. **(A–H)** Relative mRNA expression levels of **(A)** IL-1β, **(B)** IL-6, **(C)** TNFβ1, **(D)** TNFSF15, **(E)** ECE-1, **(F)** ET-1, **(G)** VEGF, and **(H)** MCP-1 in the retina of normal chow (NC) and HFD-fed mice (n=6). Gene expression was normalized to an internal control. Data are presented as mean ± standard deviation (SD). Statistical significance was determined using an unpaired t-test. ****p < 0.0001.

To evaluate the impact of a HFD on retinal tissue, we analyzed the mRNA expression levels of multiple inflammatory and vascular factors in the retinas of HFD‐fed mice and NC controls. As shown in the figure, HFD‐fed mice exhibited significantly higher retinal expression of the pro‐inflammatory cytokines IL‐1β, IL‐6, TNFβ1, and TNFSF15 (**Figures 2A-D**), suggesting an enhanced inflammatory state in the retina. In addition, we observed marked upregulation of ECE‐1 and ET‐1 (**Figures 2E, F**), key mediators involved in vasoconstriction and endothelial dysfunction. Furthermore, the pro‐angiogenic factor VEGF and the chemokine MCP‐1 (**Figures 2G, H**) were also significantly elevated in HFD‐fed mice compared to NC controls. Taken together, these findings indicate that chronic high‐fat feeding induces a pronounced inflammatory and pro‐angiogenic environment in the retina, potentially contributing to the pathogenesis of HFD‐related retinal dysfunction.

### Elevated total cholesterol positively correlates with retinal ECE‐1 and ET‐1 in HFD‐fed mice

**Figure 3:**
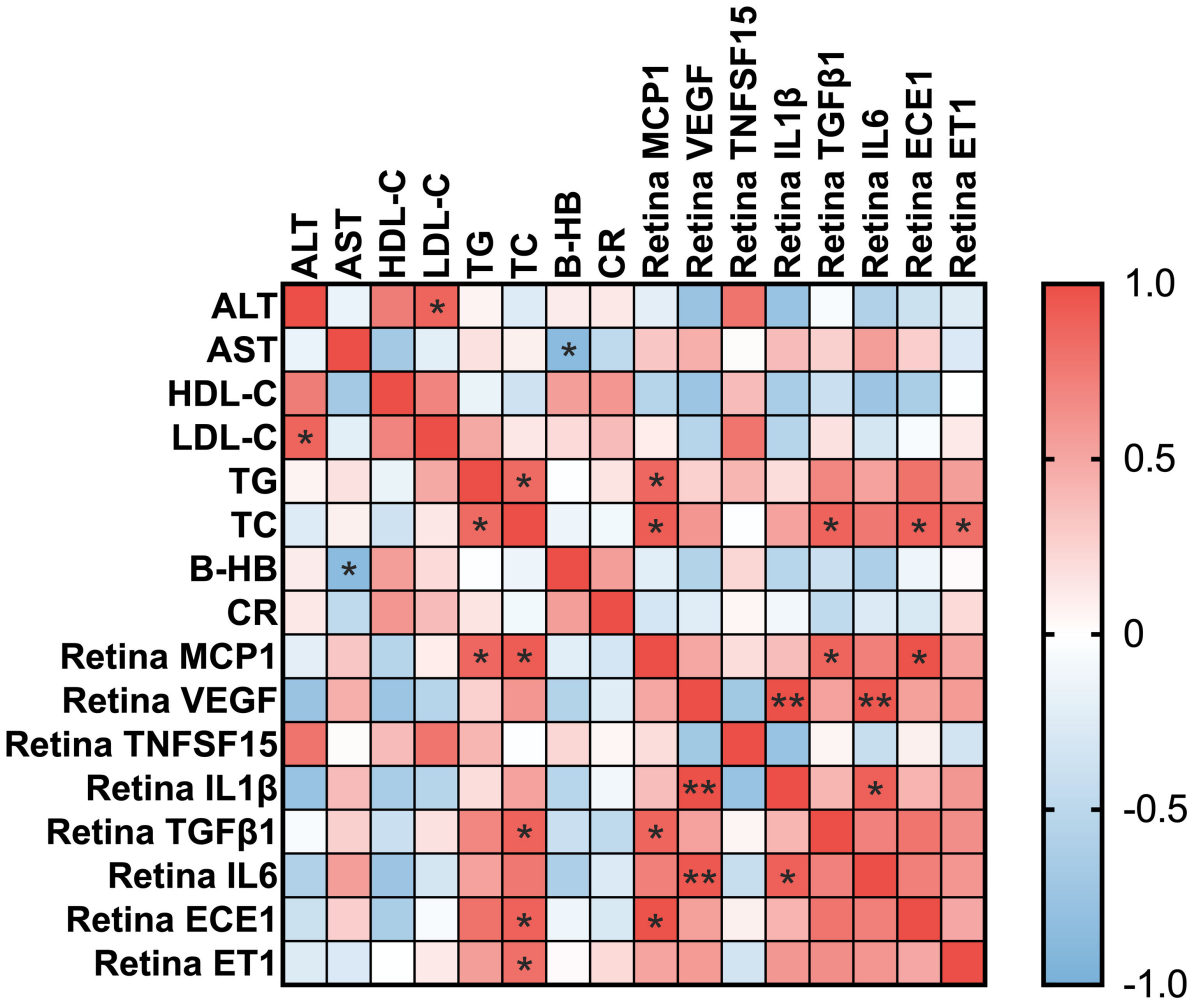
Correlation heatmap between blood parameters and retinal mRNA expression levels. Heatmap showing Pearson correlation coefficients between blood biochemical parameters and retinal inflammatory and vascular-related gene expression. Blood parameters include alanine aminotransferase (ALT), aspartate aminotransferase (AST), high-density lipoprotein cholesterol (HDL-C), low-density lipoprotein cholesterol (LDL-C), triglycerides (TG), total cholesterol (TC), β-hydroxybutyrate (B-HB), and creatinine (CR). Retinal markers include MCP-1, VEGF, TNFSF15, IL-1β, TGFβ1, IL-6, ECE-1, and ET-1. The color scale represents correlation values, with red indicating a positive correlation and blue indicating a negative correlation. * for p<0.05 and ** for p<0.01.

The correlation heatmap (**Figure 3**) illustrates the strong positive associations between blood TC levels and the retinal expression of both ECE‐1 and ET‐1. Specifically, ECE‐1 mRNA levels were significantly correlated with TC (Pearson’s r = 0.888, 95% CI: 0.2735 to 0.9877, p = 0.018*), and ET‐1 mRNA levels showed a similarly strong correlation with TC (Pearson’s r = 0.815, 95% CI: 0.008814 to 0.9790, p = 0.048*). These findings reinforce the notion that systemic dyslipidemia, particularly elevated total cholesterol, is closely linked to upregulated vasoconstrictive and inflammatory pathways in the retina. Taken together, our data provide compelling evidence for a mechanistic link between high‐fat diet‐induced metabolic disturbances and retinal pathology, highlighting ECE‐1 and ET‐1 as critical mediators in this process.

## Materials and methods

### Animal model and diets

A total of 12 male C57BL/6 mice (5–6 weeks old) were obtained from HFK Bioscience Co. Ltd. (Beijing, China) and housed under standard laboratory conditions (12-hour light/dark cycle, 23 ± 2°C) with unrestricted access to water. Afer one-week acclimation period, mice were randomly assigned to either a NC group (n=6) or a HFD group (n=6) using a computer-generated randomization sequence to ensure balanced allocation. The HFD group was fed a specialized high-fat diet (D12492, Research Diets, New Brunswick, USA) for 15 weeks, while the NC group received a standard chow diet. Body weight was recorded weekly throughout the experimental period. This study was performed in accordance with relevant guidelines and regulations. All methods were reported in accordance with ARRIVE guidelines and were approved by the Institutional Animal Care and Use Committee (IACUC) of the Chu Hsien-I Memorial Hospital of Tianjin Medical University (Approval No. DXBYY-IACUC-2021038).

### Serum biochemical analysis

Fasting blood samples were collected and centrifuged to isolate serum, which was stored at −80 °C until analysis. Levels of AST, ALT, CR, TC, TG, LDL-C, and HDL-C were measured using commercial assay kits (Biosino Bio-Technology Inc., Beijing, China). To assess ketone metabolism, B-HB was quantified in non-fasting blood samples using an ELISA kit (Meimian Industrial Co. Ltd., Jiangsu, China).

### Quantitative real time PCR

Total RNA was extracted from retinal tissues using TRIzol reagent (TaKaRa, Japan), and RNA concentration was determined spectrophotometrically. Complementary DNA (cDNA) synthesis was performed with the RevertAid First Strand cDNA Synthesis Kit (Thermo Fisher Scientific, USA). qRT-PCR was conducted using TB Green^®^ Premix Ex Taq™ II (Tli RNaseH Plus) on a Roche LightCycler^®^ 480 II system. Amplification conditions consisted of an initial denaturation at 95 °C for 5 minutes, followed by 40 cycles of 95 °C for 10 seconds and 60 °C for 30 seconds. Gene expression was normalized to GAPDH as an internal reference, and relative quantification was performed using the ΔΔCt method. The sequences of the primers used in this study are listed in **Table 1**. qPCR analyses were performed on retinal samples from all mice (n=6 per group, serving as biological replicates). Each biological replicate was assayed in triplicate (technical replicates) to ensure reliability. Data were averaged across technical replicates for each sample prior to statistical analysis.

**Table 1:** Mouse primers used for qRT-PCR.

Gene	Primer
GAPDH	F: GAGCCAAAAGGGTCATCATCT
	R: AGGGGCCATCCACAGTCTTC
MCP-1	F: GCTGACCCCAAGAAGGAATG
	R: TGAGGTGGTTGTGGAAAAGG
ECE-1	F: TCATCGGCTCGCTCTCCAACT
	R: CCTTACCAGACTTCGCACTTGTGA
ET-1	F: TGACGCACAACCGAGCACATT
	R: GGGAACACCTCAGCCTTTCTTGG
IL6	F: TTGCCTTCTTGGGACTGATG
	R: ACTCTTTTCTCATTTCCACGATTT
VEGF	F: GAAGACACGGTGGTGGAAGAAGAG
	R: GGGAAGGGAAGATGAGGAAGGGTA
TNFSF15	F: TGGTGGTCACATCTCTGGCTTCAT
	R: TGGGCAGGGCTCTCAGGTTTG
IL1β	F: TCACAAGCAGAGCACAAGCC
	R: CATTAGAAACAGTCCAGCCCATAC
TGF-β1	F: ATTCCTGGCGTTACCTTGG
	R: AGCCCTGTATTCCGTCTCCT

## Discussion

In this study, we demonstrated that HFD induces systemic metabolic disturbances, characterized by obesity, dyslipidemia, and hepatic stress, and that these disturbances correlate with significant alterations in retinal gene expression. Notably, we identified strong positive associations between TC levels and the upregulation of both ECE‐1 and ET‐1 in the retina. Our findings support the association between systemic dyslipidemia—particularly elevated TC—and retinal endothelial dysfunction and inflammation, potentially involving the ECE‐1/ET‐1 axis. This discussion aims to integrate our results with existing literature, highlight potential mechanisms, and propose future directions for research.

Our data confirm that HFD feeding is an effective approach for modeling obesity and dyslipidemia in mice. Consistent with numerous previous studies, HFD‐fed mice rapidly gained weight, exhibited higher levels of liver enzymes (ALT, AST), and displayed disrupted lipid profiles (increased TC, LDL‐C, HDL‐C) relative to NC controls. These systemic alterations reflect the multifactorial impact of HFD, which not only elevates caloric intake but also perturbs metabolic pathways regulating lipid and glucose homeostasis (12). Obesity and dyslipidemia are intimately linked to insulin resistance, hepatic steatosis, and chronic low‐grade inflammation—features collectively associated with metabolic syndrome (13).

Our results showed no significant changes in serum TG between HFD and NC groups, which might appear surprising given the well‐documented hypertriglyceridemia often observed in obese or diabetic states. However, several factors can account for this finding. First, TG levels can vary considerably depending on the duration of HFD feeding, the time of blood sampling (fasted versus fed state), and the specific composition of the diet (14). Second, some rodent models adapt by storing excess lipids in adipose tissue rather than maintaining high circulating TG levels, leading to obesity with normal or moderately elevated serum TG (15). Thus, while elevated TG is a hallmark in many human patients with metabolic syndrome, rodent models can exhibit a more complex interplay among hepatic lipid production, peripheral uptake, and storage. Moreover, while HFD feeding induced significant elevations in total cholesterol and hepatic stress markers, triglyceride levels remained unchanged compared to controls. This may be attributable to the specific composition of the D12492 HFD (60% kcal from fat, primarily lard), which is known to preferentially elevate cholesterol over triglycerides in C57BL/6 mice, as opposed to diets with higher carbohydrate content that exacerbate hypertriglyceridemia (16). Strain-specific metabolic responses could also play a role, with C57BL/6 mice exhibiting greater susceptibility to cholesterol dysregulation. Despite unchanged triglyceride levels, the strong correlation between TC and retinal ECE-1/ET-1 expression raises the possibility that cholesterol-related mechanisms, such as oxidized LDL accumulation, may play a more prominent role in early retinal changes under high-fat conditions. This underscores the potential relevance of lipid-lowering strategies in mitigating metabolic retinal stress.

Our investigation into the retinal effects of HFD revealed marked upregulation of pro‐inflammatory cytokines (IL‐1β, IL‐6, TNFβ1, and TNFSF15) and pro‐angiogenic factors (VEGF, MCP‐1). These findings mirror the inflammatory milieu described in diabetic retinopathy (DR) and other retinopathies driven by metabolic dysregulation. Inflammation in the retina is multifaceted, involving activation of microglia, infiltration of macrophages, and the release of pro‐inflammatory mediators that can disrupt the blood‐retinal barrier (BRB) (17). Moreover, chronic inflammation stimulates endothelial cells to produce VEGF, which promotes pathological neovascularization, a hallmark of advanced DR (18). The observation that MCP‐1 is significantly elevated in HFD‐fed mice underscores the role of chemokines in retinal pathology. MCP‐1 is a potent chemoattractant for monocytes and other immune cells, contributing to a pro‐inflammatory cascade that exacerbates tissue damage (19). Similarly, TNFSF15, also known as TL1A, has been implicated in various inflammatory diseases, and its upregulation in the retina may promote endothelial cell activation and angiogenesis (20). The interplay among these cytokines and chemokines sets the stage for a vicious cycle of retinal injury, where inflammation begets further inflammation, ultimately impairing visual function.

One of the most striking findings in our study is the pronounced increase in retinal ECE‐1 and ET‐1 expression in HFD‐fed mice. ET‐1 is widely recognized as one of the most potent vasoconstrictors, exerting powerful effects on vascular tone, cell proliferation, and inflammatory signaling. In physiological conditions, ET‐1 plays a role in maintaining basal vascular tone; however, under pathological conditions such as diabetes, hypertension, or hyperlipidemia, its levels can become aberrantly high. ECE‐1, the enzyme that catalyzes the conversion of big ET‐1 to active ET‐1, represents a critical control point in ET‐1 biosynthesis (7, 8). Our data demonstrate a positive association between elevated TC and increased retinal ECE‐1 and ET‐1 expression, suggesting that systemic lipid overload may contribute to ET‐1‐related vasoregulatory and inflammatory alterations in the retina.

Mechanistically, dyslipidemia may induce oxidative stress and endothelial dysfunction, which in turn upregulate ECE‐1 transcription and ET‐1 production. ET‐1 can bind to endothelin receptors (ET_A and ET_B) on endothelial cells, smooth muscle cells, and pericytes, eliciting potent vasoconstrictive and pro‐inflammatory signals. Chronic ET‐1 overproduction disrupts the delicate balance between vasodilatory and vasoconstrictive factors in the retina, compromising blood flow, promoting capillary dropout, and accelerating microvascular damage (21). These processes are integral to the pathogenesis of DR, suggesting that HFD‐induced hypercholesterolemia may recapitulate some features of diabetic retinopathy through the same molecular pathways.

The positive correlation between TC and retinal ECE‐1/ET‐1 expression underscores a direct link between systemic lipid dysregulation and local vascular changes. Hypercholesterolemia is often considered an independent risk factor for microvascular complications, including retinopathy. Interventions that lower plasma LDL‐C and total cholesterol, such as statin therapy, have been associated with improved retinal outcomes in some patients (22). Although our study did not assess the therapeutic impact of lipid‐lowering agents, the robust correlations observed here imply that controlling cholesterol levels might mitigate retinal damage.

From a clinical perspective, our HFD model shares several similarities with DR, a leading cause of vision loss in metabolic disorders, including upregulation of pro-inflammatory cytokines (e.g., IL-1β, IL-6, TNF-α) and vascular factors like ET-1, which contribute to endothelial dysfunction and microvascular leakage in both conditions (23, 24). For instance, elevated ET-1 in DR promotes vasoconstriction and ischemia, mirroring our observations in HFD-fed retinas. However, key differences exist: DR is typically driven by hyperglycemia and advanced glycation end-products, whereas our model emphasizes dyslipidemia without overt diabetes (e.g., no significant glucose elevations were noted). Thus, the HFD model may better represent obesity-related retinal changes independent of hyperglycemia, such as those in non-diabetic metabolic syndrome. These parallels suggest that ECE-1/ET-1 inhibitors could have therapeutic potential in both DR and diet-induced retinopathies, warranting further comparative studies.

This study has several limitations. First, while our analyses revealed robust gene expression changes via qPCR, we did not perform functional validation—such as electroretinography (ERG), fundus imaging, retinal histology, or fluorescein angiography—which would help confirm the impact of these molecular alterations on retinal structure and function. Second, although we evaluated systemic lipid profiles, we did not conduct retinal-specific lipidomics, potentially missing localized lipid disruptions that may directly influence retinal health. Third, the 15-week HFD exposure models subacute metabolic stress and was sufficient to induce obesity and dyslipidemia; however, longer studies are needed to capture chronic retinal changes such as microaneurysms, pericyte dropout, or neovascularization, which are relevant to late-stage retinopathy. Fourth, while we found a strong correlation between total cholesterol and retinal ECE-1/ET-1 upregulation, the underlying molecular mechanisms remain unexplored. Pathways involving oxidative stress, nuclear factor kappa B (NF-κB), or other transcriptional regulators could be involved and warrant further investigation. Fifth, as with all animal models, species differences in lipid metabolism and retinal anatomy may limit the direct clinical translatability of our findings. Future clinical studies are necessary to validate whether similar mechanisms occur in humans. Sixth, the specific composition of the D12492 HFD—high in saturated fat from lard—may not fully reflect the diversity of human dietary patterns. Therefore, testing alternative diet formulations could provide a broader understanding of how various lipid profiles influence retinal pathology. Additionally, our gene expression analyses were performed on whole retinal tissue, which contains a heterogeneous mix of neurons, glia, and vascular cells. As such, we cannot determine which specific cell types contributed to the observed upregulation of inflammatory and vasoregulatory genes such as ECE-1 and ET-1. Finally, some of our correlation coefficients showed broad confidence intervals, indicating potential variability due to limited sample size and underscoring the need for replication in larger cohorts. Future studies using cell type–specific techniques like single-cell RNA sequencing or *in situ* hybridization are warranted to clarify the cellular sources and functional implications of these molecular changes.

Our study provides compelling evidence that HFD‐induced dyslipidemia is intimately connected to retinal inflammation and endothelial dysfunction, mediated in part by the ECE‐1/ET‐1 axis. Elevated TC levels strongly correlate with increased ECE‐1 and ET‐1 expression in the retina, suggesting that lipid overload can directly trigger vasoconstrictive and pro‐inflammatory pathways. Furthermore, the pronounced upregulation of inflammatory cytokines and angiogenic factors in the retina underscores the multifactorial nature of HFD‐driven retinal pathology, encompassing not only endothelial injury but also immune cell recruitment and cytokine release. These findings expand the current understanding of how systemic metabolic stress translates into local ocular complications. They also open the door to novel therapeutic strategies targeting ECE‐1/ET‐1 or cholesterol metabolism in the context of retinopathy prevention and treatment. Ultimately, clarifying the molecular mechanisms that link dyslipidemia to retinal damage may enable more effective interventions to preserve visual function in individuals at risk for metabolic diseases.

## Conclusions

In summary, our work highlights the importance of comprehensive metabolic management, encompassing both glycemic control and lipid regulation, in safeguarding retinal health. By shedding light on the ECE‐1/ET‐1 axis as a key molecular conduit between systemic dyslipidemia and retinal vascular pathology, we propose a new framework for understanding and potentially mitigating the ocular consequences of metabolic syndrome and related conditions.

## Data Availability

The raw data supporting the conclusions of this article will be made available by the authors, without undue reservation.
