# Correction: High-fat diet-induced dyslipidemia drives retinal ECE-1 and ET-1 upregulation

**DOI:** 10.3389/fendo.2025.1699321

**Published:** 2025-09-16

**Authors:** Shuo Sun, Huilan Zhang, Jing Chen, Kaiwen Hei, Yi Hou, Yanhui Yang, Chunyan Shan, Longli Zhang

**Affiliations:** ^1^ Tianjin Key Laboratory of Retinal Functions and Diseases, Tianjin Branch of National Clinical Research Center for Ocular Disease, Eye Institute and School of Optometry, Tianjin Medical University Eye Hospital, Tianjin, China; ^2^ NHC Key Laboratory of Hormones and Development, Chu Hsien-I Memorial Hospital and Tianjin Institute of Endocrinology, Tianjin Medical University, Tianjin, China; ^3^ Tianjin Key Laboratory of Metabolic Diseases, Tianjin Medical University, Tianjin, China

**Keywords:** high-fat diet, endothelin-1, endothelin-converting enzyme-1, retina, dabetic retinopathy

Author “Longli Zhang” was erroneously assigned as a single corresponding author. The correct corresponding authors are “Longli Zhang, zhanglonglieye@126.com and Chunyan Shan, chunyanshan@hotmail.com”.

The original version of this article has been updated.

